# Deep Learning Applications in Dental Image-Based Diagnostics: A Systematic Review

**DOI:** 10.3390/healthcare13121466

**Published:** 2025-06-18

**Authors:** Osama Khattak, Ahmed Shawkat Hashem, Mohammed Saad Alqarni, Raha Ahmed Shamikh Almufarrij, Amna Yusuf Siddiqui, Rabia Anis, Shahzad Ahmad, Muhammad Amber Fareed, Osama Shujaa Alothmani, Lama Habis Samah Alkhershawy, Wesam Waleed Zain Alabidin, Rakhi Issrani, Anshoo Agarwal

**Affiliations:** 1 Department of Restorative Dentistry, College of Dentistry, Jouf University, Sakaka 72311, Saudi Arabia; dr.osama.khattak@jodent.org; 2 Oral Medicine and Periodontology, Faculty of Dentistry, Damanhour University, Damanhur 22522, Egypt; dr.ahmed.shawkat75@gmail.com; 3 Department of Oral & Maxillofacial Surgery and Diagnostic Sciences, College of Dentistry, Jouf University, Sakaka 72311, Saudi Arabia; dr.mohammed.alqarni@jodent.org; 4 College of Dentistry, Jouf University, Sakaka 72311, Saudi Arabia; raha.shamikh.almufarrij@jodent.org (R.A.S.A.); wesam.waleed.alabidin@jodent.org (W.W.Z.A.); 5 Department of Endodontics, Faculty of Dentistry, King Abdulaziz University, Jeddah 22230, Saudi Arabia; asiddiqui@kau.edu.sa (A.Y.S.);; 6 Department of Health Professions Education, Isra Dental College, Isra University, Hydrabad 73000, Pakistan; dr.rabi87@gmail.com; 7 Faculty of Medicine and Health Science, The University of Buckingham, Buckingham MK18 1EG, UK; shahzad.ahmad@buckingham.ac.uk; 8 Clinical Sciences Department, College of Dentistry, Ajman University, Ajman 346, United Arab Emirates; 9 Centre of Medical and Bio-Allied Health Sciences Research, Ajman University, Ajman 346, United Arab Emirates; 10 Department of Preventive Dentistry, College of Dentistry, Jouf University, Sakaka 72311, Saudi Arabia; 11 Department of Pathology, Northern Border University, Arar 91431, Saudi Arabia; dranshoo30@gmail.com

**Keywords:** artificial intelligence, diagnosis, machine learning, dental sciences, AI models

## Abstract

**Background**: AI has been adopted in dentistry for diagnosis, decision making, and therapy prognosis prediction. This systematic review aimed to identify AI models in dentistry, assess their performance, identify their shortcomings, and discuss their potential for adoption and integration in dental practice in the future. **Methodology**: The sources of the papers were the following electronic databases: PubMed, Scopus, and Cochrane Library. A total of 20 out of 947 needed further studies, and this was encompassed in the present meta-analysis. It identified diagnostic accuracy, predictive performance, and potential biases. **Results**: AI models demonstrated an overall diagnostic accuracy of 82%, primarily leveraging artificial neural networks (ANNs) and convolutional neural networks (CNNs). These models have significantly improved the diagnostic precision for dental caries compared with traditional methods. Moreover, they have shown potential in detecting and managing conditions such as bone loss, malignant lesions, vertical root fractures, apical lesions, salivary gland disorders, and maxillofacial cysts, as well as in performing orthodontic assessments. However, the integration of AI systems into dentistry poses challenges, including potential data biases, cost implications, technical requirements, and ethical concerns such as patient data security and informed consent. AI models may also underperform when faced with limited or skewed datasets, thus underscoring the importance of robust training and validation procedures. **Conclusions**: AI has the potential to revolutionize dentistry by significantly improving diagnostic accuracy and treatment planning. However, before integrating this tool into clinical practice, a critical assessment of its advantages, disadvantages, and utility or ethical issues must be established. Future studies should aim to eradicate existing barriers and enhance the model’s ease of understanding and challenges regarding expense and data protection, to ensure the effective utilization of AI in dental healthcare.

## 1. Introduction

Artificial intelligence (AI) is transforming dentistry in that early diagnosis is more accurate with advanced treatment planning, treatment coordination, and patient monitoring. Envisaged as the science of creating systems capable of emulating intelligent human actions, AI has undergone extensive deployment across the sphere of healthcare, and dentistry is no exception. With the help of AI technologies, dentists are able to easily analyze various forms of imaging, make better patient-specific treatment decisions, and enhance patient care [1,2].

AI holds a large potential in dentistry, and one of the best areas where it could be applied is medical image analysis. For instance, a sub-type of AI known as convolutional neural networks (CNNs) is used in the diagnosis of dental caries, apical lesions, and bone loss from periapical radiographic images as well as panoramic images [3]. Such models can independently assess affected teeth and separate various structures in the mouth to identify regions that require treatment to minimize diagnostic mistakes and time consumption [4,5]. Prior studies have shown that CNNs have a higher diagnostic accuracy than conventional approaches for detecting carious lesions, and periapical radiographs offer proper image clarity for caries [6].

Other than diagnostics, there is the application of AI in treatment procedures. Current dental operating systems are now fully automated using robotic systems that have incorporated AI, and these systems can now perform 3D tooth preparations without destroying the surrounding tissues [7,8]. In the context of restorative dentistry, ANNs assess material characteristics to avoid the debonding process of composite restorations [9]. Similarly, orthodontic treatments have been on the receiving end, with applications such as cephalometric analyses and planning adjustments to harmonize facial aesthetics [10].

It is concluded that there are great opportunities in the application of AI, but there are also several problems with AI in the field of dentistry. Some of these issues include patient data confidentiality, the ability to obtain patient consent, and potential algorithmic bias, which must be addressed to minimize or eliminate bias and unfair treatment in the equitable sharing of these services [11]. Moreover, the reliability of AI systems depends on the quality and diversity of the training datasets, which hinders the systems’ applicability outside researched settings. The high cost of integrating AI may be a challenge for small firms, particularly in explaining why the implementation of advanced dental technologies may lead to unequal distribution [12].

AI application in dentistry is fundamental because it changes the dental field in a way that makes care data-driven, accurate, and timely. It is proficient in the study of big data and displaces arduous jobs that burden clinicians while enhancing the lives of patients [2,13]. Nevertheless, to unlock the full potential of AI as a resource, much attention has to be paid to AI’s limitations, both ethical and technical, as well as to its availability for a wider range of use. Future advancement in modern dentistry will be premised on findings derived from focused studies exploring the applicability of AI in dental practice [1].

## 2. Materials and Methods

This review has been registered in the INPLASY database with the registration number INPLASY202530022, and the associated DOI is 10.37766/inplasy2025.3.0022.

### 2.1. Methodological Framework

This review adhered to the PRISMA guidelines, formulated to address the research question:

*“Which types of AI approaches are applied in dentistry and to what extent and in what way is AI improving the diagnosis in dentistry, the quality of decisions made and the outcomes of dental procedures?”*

*PICOS, which represents population, intervention, comparison, outcome, and study design, was utilized in the formulation of this investigation.*

CAD/CAM, computerized representations of the clinic, apical, bitewing, orthopantomographic, or CBCT radiographs, and 2D and 3D patient and simulator facial images were all part of the package. The primary focus was on the application of robotics, natural language processing and deep learning, in dental care evaluation, therapy, and prognosis. The outcomes measured included accuracy, efficiency, support for the critical conclusion, the area under the curve and applications of AI in various specialties of dentistry.

### 2.2. Search Strategy

To assess machine learning dental applications, as well as neural networks and their role in oral care, we searched databases such as PubMed, Scopus and the Cochrane Library for studies published between 2014 and 2023. The search terms used were (“Artificial Intelligence in dentistry” OR “AI in dentistry”) AND (“Machine Learning”) AND (“Neural Networks”) AND (“Dental”) AND (“Oral Health”).

As part of the search process, Boolean operators such as AND and OR were used to remove any inconsequential results. For example, when entering the search query “AI in dentistry” and “machine learning”, this helped to narrow down the results to show only the studies related to neural networks used in dentistry.

**Figure 1:**
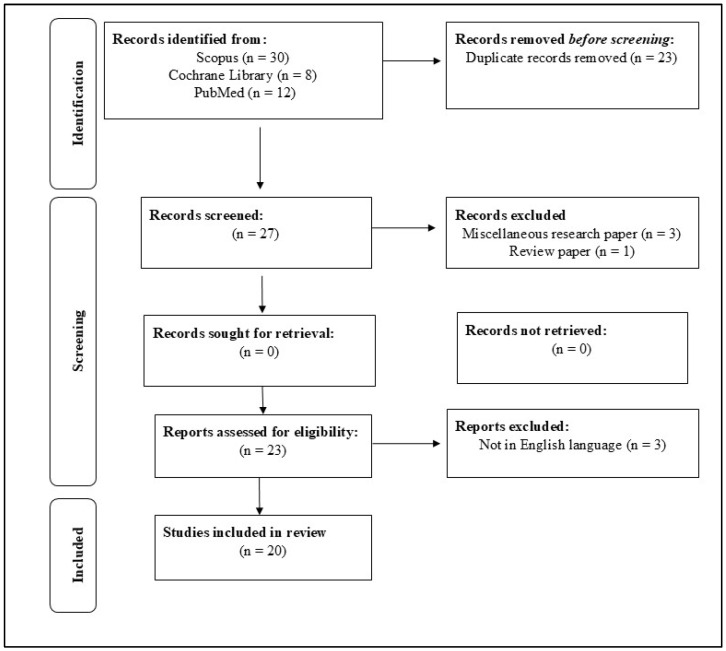
The PRISMA methodology was employed for identifying pertinent studies for this study.

Inter-rater agreement was assessed using Cohen’s kappa coefficient to ensure consistency during study selection and data extraction. The kappa value was calculated at 0.82, indicating strong agreement. Disagreements between the two primary reviewers (1st and 3rd author) were resolved through discussion, and unresolved discrepancies were referred to a third reviewer (12th author). The review hence included 20 papers that passed the inclusion criteria as depicted in [Figure 1], a PRISMA flow diagram.

### 2.3. Criteria of Exclusion and Inclusion

#### 2.3.1. Criteria for Inclusion

1. Studies along with specified keywords;
2. Articles (research) available from 2014 to 2023;
3. English-language studies; and
4. Original research articles.

#### 2.3.2. Criteria for Exclusion

1. Studies not focusing on AI-based diagnostics in pediatric dentistry;
2. Articles not including full-text access; and
3. Ongoing research projects.

Pediatric dentistry focuses on children who have unique dental conditions and diagnostic needs. AI diagnostics in pediatric dentistry uses specific tools and techniques for children that may not apply to other groups. Including pediatric research could increase dataset diversity, complicating synthesis. The diagnostic challenges in children’s dentistry, including behavior management and limited data, differ significantly from those in adult dentistry, making comparisons less meaningful.

For precise results, two reviewers independently performed the data extraction. The reviewers obtained details regarding the studies and their outcomes. After the extraction, notes were compared to check for any discrepancies, and a consensus was reached in cases where the notes differed.

### 2.4. Quality Assessment

The appraisal of the studies that were selected was conducted using The Cochrane Handbook for Systematic Reviews of Interventions (v5.1.0) criteria [11]. In addition to this, the AMSTAR 2 checklist was used as a complementary tool to enhance the methodological rigor and transparency of this systematic review.

The QUADAS-2 framework was adapted to assess risk of bias for diagnostic AI models. Assessment criteria included randomization, blinding, dropout rates, the accuracy of outcome variables, sample size estimation, and examiner reliability.

## 3. Results

Thus, the search was conducted using pre-coordinated terms and Boolean connectors such as AND, resulting in 50 articles. After removing 23 articles that were similar, 27 articles needed further scrutiny. Following the inclusion and exclusion criteria, 20 relevant studies were selected for this systematic review.

Data extraction focused on capturing critical details from each study, including the authors’ names and publication year, input data sources, study objectives, AI models employed, and resulting outcomes. A detailed summary of these studies is presented in Table 1, which encapsulates essential information such as the study design, AI methodologies applied, their practical applications, evaluation metrics, and significant findings. The present tabulation provides a detailed outlook of AI practices in dentistry and their impact on diagnostic efficiency and decision making in therapeutic care.

**Figure 2:**
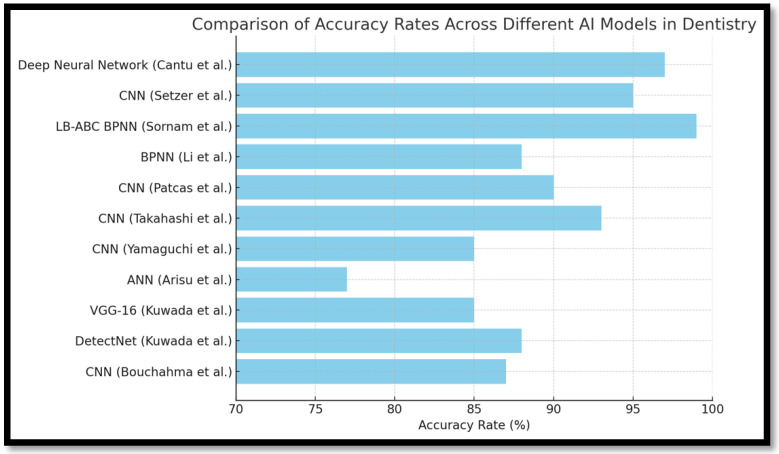
Comparison of accuracy rates across different AI models in dentistry. (Cantu et al. [14] (2020), Setzer et al. [15] (2020), Sornam et al. [16] (2019), Li et al. [8] (2020), Patcas et al. [12] (2019), Takahashi et al. [7] (2020), Yamaguchi et al. [11] (2019), Arisu et al. [6] (2018), Kuwada et al. [10] (2020), Bouchahma et al. [4] (2019)).

**Figure 3:**
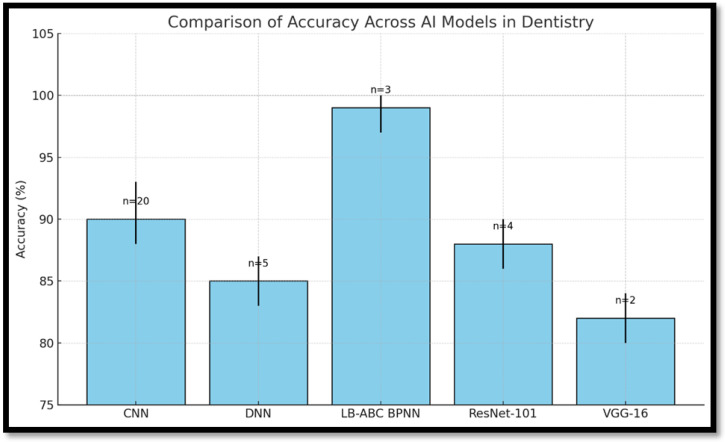
A comparison of accuracy rates across different AI models in dentistry. (Error bars represent confidence intervals where available. The sample size (n), indicated above each bar, reflects the number of studies or datasets contributing to each model’s performance assessment).

Figure 2 reveals that CNNs perform consistently across diagnostic tasks with an accuracy range of 85–93%, making them the most reliable for image-intensive applications. In contrast, the LB-ABC BPNN model, though evaluated in fewer studies, exhibited exceptional performance with near-perfect classification accuracy in certain tasks, suggesting suitability for complex, non-image-based diagnostic decisions. Figure 3 confirms these observations but also highlights that models like VGG-16 and ResNet-101, while promising, lack broad external validation, limiting current clinical applications.

**Table 1:** A comprehensive report of the studies that were included.

Study (Author, Year)	Study Design	AI Model	Dataset Type and Size (N)	Preprocessing Techniques	Validation Method	Application	Experimental Method	Outcomes
Bouchahma et al., [4] (2019)	Clinical study	CNN	Panoramic X-rays, N = 700	Grayscale conversion, normalization	Not reported	Operative dentistry and Endodontia	Deep Learning → Supervised Learning → CNN	Accuracy: 87%; RCT detection: 88%; Fluoride: 98%
Arisu et al., [6] 2018	Clinical study	ANN	Intraoral images, N = 2400	Not reported	Internal testing only	Restorative dentistry	Deep Learning → Supervised Learning → ANN	Composite curing prediction; No metric reported
Takahashi et al., [7] 2020	Experimental study	CNN	Panoramic X-rays, N = 1498	Image enhancement, segmentation	Train/test split	Prosthodontics	Deep Learning → Supervised Learning → CNN	Qualitative classification; No metric reported
Li et al., [8] 2020	Clinical study	Automated photo analysis	Facial and intraoral photos, N = 1050	Alignment of landmarks	Manual comparison	Esthetic dentistry	Image Analysis → Automated Integration → Not specified	Subjective cosmetic application; No metric reported
Kuwada et al., [10] 2020	Clinical study	Detect Net, Alex Net, and VGG-16	Panoramic X-rays, N = 550	Grayscale conversion, normalization	Train/validation/test split	Orthodontics	Deep Learning → Supervised Learning → CNN (DetectNet, etc.)	Precision, recall, F1-score reported
Yamaguchi et al., [11] 2019	Clinical study	CNN	Cephalometric radiographs, N = 1146	Image resizing, grayscale conversion	Holdout validation	Restorative dentistry	Deep Learning → Supervised Learning → CNN	Prediction of crown debonding; No metric reported
Patcas et al., [12] 2019	Study of Cohort	CNN	Orthodontic photos, N = 1200	Image cropping, standardization	Internal dataset	Orthodontics	Deep Learning → Supervised Learning → CNN	Subjective age/attractiveness estimation; No metric reported
Li et al., [13] (2015)	Experimental research	GA and BPNN	Orthodontic measurements, N = 1000	Feature normalization	Internal validation	Aesthetic dentistry	Deep Learning → Supervised Learning → GA + BPNN	Objective tooth color matching; No metric reported
Kositbowornchai et al. [17] (2016)	Clinical study	LVQ Neural Network	Panoramic X-rays, N = 600	Image normalization	Holdout validation	Restorative dentistry	Deep Learning → Supervised Learning → LVQ-NN	Caries detection; No metric reported
Patcas et al., [18] (2019)	Clinical study	CNN	Orthodontic photographs, N = 1200	Image cropping, standardization	Internal dataset; no k-fold	Orthodontics	Deep Learning → Supervised Learning → CNN	Assessment of cleft patient profiles and frontal aesthetics; No metric reported
Vranckx et al., [19] (2020)	Clinical study	CNN and ResNet-101	Dental radiographs, N = 3000+	Rescaling, augmentation	Cross-validation not specified	Operative dentistry	Deep Learning → Supervised Learning → CNN (ResNet-101)	Molar segmentation; No metric reported
Lee et al., [20] (2020)	Clinical study	ML (e.g., Decision Trees, SVM)	TMJ radiographs, N = 2100	Grayscale conversion, filtering	Train/test split	Oral and Maxillofacial Surgery	ML → Supervised Learning → SVM, Decision Trees	TMJOA classification; Accuracy: 95%
Cui et al., [21] (2020)	Cohort study	CDS ML model	Dataset from 5 hospitals, N = ~4000 cases	Data normalization cleaning	Separate test set	Oral and Maxillofacial Surgery	ML → Supervised Learning → CDS model	Accuracy: 99.16%
Sornam and Prabhakaran [16] (2019)	Clinical study	BPNN with LB-ABC	Dental records, N = 750	Data normalization	10-fold cross-validation	Restorative dentistry	Deep Learning → Supervised Learning → Hybrid BPNN	Accuracy: 99.16%
Setzer et al., [15] (2020)	Clinical study	DL for CBCT	CBCT scans, N = 1000	Noise removal, segmentation	External validation	Endodontics	Deep Learning → Supervised Learning → CNN	Lesion detection; No metric reported
Cantu et al., [14] (2020)	Clinical study	CNN	CBCT images, N = 800	Voxel normalization, filtering	Stratified train/test split	Operative dentistry and Oral Radiology	Deep Learning → Supervised Learning → CNN	Performance exceeds clinicians; No metric reported
Aliaga et al., [22] (2020)	Experimental study	Automatic segmentation	Facial scans, N = 350	Mesh alignment, surface smoothing	Manual comparison	Operative dentistry and Oral and Maxillofacial Surgery	Image Analysis → Automated → Not specified	Osteoporosis detection; No metric reported
Kim et al., [23] (2018)	Case–control study	ML classifiers (SVM, RF, etc.)	Dental images, N = 2500	Feature scaling	Train/test split	Oral and Maxillofacial Surgery and Oral Medicine	ML → Supervised Learning → SVM, RF	BRONJ prediction; Accuracy: 91.8%
Dumast et al., [24] (2018)	Case–control study	CNN and Shape variation	Radiographs, N = 1820	Histogram equalization	Test set validation	Oral and Maxillofacial Surgery	Deep Learning → Supervised Learning → CNN Bone classification; Accuracy reported	Morphological classification; Accuracy: 86.5%
Sorkhabi and Khajeh [25] (2019)	Clinical study	3D CNN	Intraoral scans, N = 1200	3D normalization	6-month clinical validation	Implant dentistry	Deep Learning → Supervised Learning → 3D CNN	Alveolar bone density classification; Accuracy: 91.2%

DL → SL → CNN: Deep learning through supervised learning using a convolutional neural network. ANN: artificial neural network; BPNN: backpropagation neural network; CBCT: Cone Beam Computed Tomography; GA: Genetic Algorithm; LVQ-NN: Learning Vector Quantization Neural Network; BRONJ: Bisphosphonate-Related Osteonecrosis of the Jaw. “No metric reported” indicates that the study described an outcome without specifying performance statistics like accuracy, precision, or recall.

For caries detection, CNNs have been very accurate with accuracy ranging to 93%. These outcomes have far exceeded the performance of typical diagnostic strategies pointing toward the higher capacity of CNNs to analyze intricate dental images and detect carious lesions effectively and accurately. Likewise, when it comes to orthodontic treatment planning, CNN models also showed very good results across three databases, with accuracy over 85%. This accuracy reaffirms the applicability of radioactivity to well-documented work, such as treatment planning and prognosis. The results reveal the ability of CNNs to revolutionize the dental practice and place CNNs as ideal candidates for implementation into clinical environments to improve diagnostic capability and decision-making rates.

Deep learning, in general, and AI in its various forms hold high degrees of promise for tackling an entire range of pathologies, and even subjects, especially in the medical field of dentistry and its branches. Such applications include threshold caries detection and diagnosis, VR identification, and apical lesion assessment, so that appropriate management can be performed on time. The ability of air systems to diagnose diseases includes salivary gland disorders, maxillofacial cysts, osteoporosis, and detection and monitoring proficiency. They are capable of detecting cancerous lesions, evaluating the degree of alveolar bone loss, and providing information necessary for cephalometric analysis, which is an important facet of orthodontic diagnosis and planning.

Moreover, AI tools have been used to diagnose potential orthodontic extractions, analysis treatment plans, and measurements important for age and sex estimations. These models apply advanced computational techniques to improve diagnostic accuracy and patient outcomes. Because of their ability to work with large volumes of information and provide impartial conclusions, specialists consider them essential for the development of dental and medical practices.

The efficacy of CNNs in such outcomes points out and depicts their roles in enhancing diagnostic accuracy and the decision-making process in dentistry in enhancing its role in contemporary dentistry. The dominance of CNNs in dental research can be attributed primarily to their strong performance in imaging-related applications, where spatial localization and edge detection are crucial. However, CNNs are less suited to tasks involving tabular clinical data, temporal trends, or natural language processing, where other AI models may be more appropriate. For example, alternative models such as ResNet-101 and VGG-16 have demonstrated competitive performance in certain scenarios. For example, ResNet-101 was used successfully for predicting molar angulations and creating segmentation maps in operative dentistry, while VGG-16 showed usefulness in identifying impacted supernumerary teeth in panoramic radiographs. Backpropagation neural networks (BPNNs), though less common in image analysis, showed high performance in non-image-based tasks such as material classification, prediction of crown debonding, and treatment outcome forecasting. A hybrid LB-ABC BPNN model has shown to outperform CNNs in classifying oral cancer lesions, indicating the potential of hybrid models in tasks involving more complex clinical and histopathological data. Moreover, DNNs, when provided with structured input features and sufficient training data, offer high versatility and depth of learning. DNNs are also found to outperform CNNs in treatment planning scenarios where spatial image features were less critical than temporal or multi-factorial data inputs. These findings suggest that while CNNs are generally superior for image-processing tasks, other architectures may be preferable depending on the specific application and the nature of the data. Therefore, AI model selection in dentistry should be tailored to the diagnostic objective, data structure, and required accuracy level to ensure optimal outcomes.

The reviewed studies, from 2014 to 2023, encompassed four primary research designs: randomized controlled trials, clinical trials, analytical cross-sectional studies, and case–control and cohort studies. The utility of AI has been identified and categorized across various subfields of dentistry, proving its effectiveness. This widespread application reflects how deeply AI is rooted across dental specialties, how it affects the existing paradigm of dentistry, and the diverse roles they play in shaping the future of this field.

The key findings from the quality assessment are summarized below:

- **Blinding**: In the context of AI model development, blinding is relevant when human annotators are involved in labeling training datasets, as knowledge of patient identity or outcome can introduce bias into the ground truth, potentially compromising model validity. Among the included studies, only Bouchahma et al. [4] explicitly reported the use of blinding during the annotation of training data, underscoring a general lack of attention to this potential bias across the reviewed literature.
- **Randomization**: Randomization was implemented in two trials [10,19].
- **Dropout Rates**: Nineteen studies addressed and reported dropout rates [4,6,7,8,10,12,13,14,15,16,17,18,19,20,21,22,23,24,25].
- **Accuracy of Research Variables**: Research variables were explicitly checked for accuracy in 20 studies [4,6,7,8,10,11,12,13,14,15,16,17,18,19,20,21,22,23,24,25].
- **Sample Size Reporting**: Details of all 20 studies provided for sample size details [4,6,7,8,10,11,12,13,14,15,16,17,18,19,20,21,22,23,24,25].
- **Inclusion and Exclusion Criteria**: Eighteen studies specified their presence and exclusion standards [4,6,7,8,10,11,12,13,14,15,16,17,18,19,22,23,24,25].
- **Examiner Dependability**: The reliability of the examiner was reported in 19 studies [4,6,7,8,10,11,12,14,15,16,17,18,19,20,21,22,23,24,25].
- **Pre-existing Results**: The findings of 16 studies were already published or well-documented [4,7,8,10,12,13,14,15,16,17,18,19,22,23,24,25].
- **Bias Risk**: Bias risk was assessed using the QUADAS-2 framework, adapted for AI diagnostic performance studies. A risk of bias at a moderate level was found in 5 studies [6,11,13,19,20], while 15 were identified as having low-quality evidence [4,7,8,10,12,14,15,16,17,18,21,22,23,24,25].

Table 2 presents an overview of the delivered trials, described in terms of methodological characteristics and outcomes.

The risk of bias using the JBI checklist was as follows: The risk of bias among analytical cross-sectional studies was 80%. The diagnostic test accuracy studies had scores of 88% suggesting a low risk of bias. Therefore, all 20 papers were included in this systematic review because they met the laid down criteria [Table 3].

As shown in Table 4, CNNs demonstrated consistently high diagnostic accuracy (85–93%) across multiple dental imaging tasks [4,10]. However, they remain less interpretable and computationally demanding. The hybrid BPNN (LB-ABC) reported the highest accuracy (~99.16%) in caries detection [16]. DNN-based models, such as the CDS system evaluated by Cui et al., also showed strong accuracy in clinical decision-making tasks involving structured datasets [21].

## 4. Discussion

AI has introduced transformative changes across multiple domains of healthcare, including dentistry, by virtue of its capacity to analyze large volumes of data and generate reliable insights. Its integration into dental practice has accelerated in recent years, significantly enhancing diagnostic accuracy, treatment planning, patient care, and administrative efficiency. Beyond clinical diagnostics, AI has contributed to improvements in patient management through tools such as appointment-scheduling systems, virtual assistants, and AI-powered chatbots that assist with common patient queries and oral health education. These technologies also show promise in mitigating dental anxiety. As AI continues to evolve, it is expected to further streamline clinical workflows, reduce human error, and contribute to more efficient and effective dental care delivery.

The studies reviewed in this paper collectively support the high diagnostic performance of AI in various dental applications. For instance, Caliskan et al. [26] demonstrated that CNNs can match expert-level accuracy in identifying submerged primary teeth from orthopantomograms. Similarly, Kilic et al. [27] illustrated how AI can expedite forensic dental identification. Zheng et al. [28] found that among various CNN architectures for pulpitis and deep caries detection, the multimodal CNN performed best. In the area of cephalometric landmark identification, Bulatova et al. [29] noted superior accuracy and efficiency using AI models compared to manual tracing. Zhao et al. [30] reported accurate assessments of adenoid hypertrophy on lateral cephalograms using AI, while Seo et al. [31] and Kim et al. [32] found that AI models could estimate cervical vertebral maturation with accuracy ranging from 90% to 93%. Likewise, Karhade et al. [33] demonstrated high precision in detecting and classifying early childhood caries. While CNNs dominate image-based tasks, models like BPNN demonstrated better classification performance in oral lesion classification [16].

Despite these advancements, several critical challenges must be addressed for AI to achieve widespread clinical adoption in dentistry. One of the foremost considerations is the trade-off between model interpretability, computational requirements, and deployment feasibility. Interpretable models like decision trees and logistic regression offer transparency and are generally more feasible for use in resource-limited settings, but they often lack the predictive power of complex models. Conversely, black-box models such as CNNs and DNNs provide superior diagnostic accuracy, especially in image-based applications, but are difficult to interpret and require substantial computational resources. Their complexity also raises concerns about accountability and patient safety. To address these concerns, interpretability tools like SHAP (Shapley Additive Explanations) and LIME (Local Interpretable Model-Agnostic Explanations) are being explored, although their integration into clinical practice remains limited [34].

Another major limitation is the lack of standardized evaluation metrics and open-access benchmark datasets in dental AI. Unlike general medical imaging, which benefits from widely recognized datasets such as MURA and CheXpert, dentistry lacks large-scale, annotated datasets to validate and compare model performance [35]. This absence hinders reproducibility, comparability, and regulatory approval. Standardized benchmarks are essential for evaluating diagnostic performance and guiding the development of reliable, generalizable AI systems.

The applicability of AI models to real-world clinical environments remains a significant concern. Most reviewed models were validated in controlled research settings using limited or demographically narrow datasets. For example, Kuwada et al. [10] reported performance metrics such as precision, recall, and F1-score for impacted tooth detection using panoramic radiographs; however, their model was only internally validated, limiting its generalizability to broader clinical settings. Similarly, Arisu et al. [6] used an ANN to predict composite curing outcomes but did not report external validation or integration into a clinical workflow. In contrast, Sornam and Prabhakaran [16] achieved a high accuracy of 99.16% using a hybrid LB-ABC BPNN model with 10-fold cross-validation, yet no evidence of clinical deployment was provided. Setzer et al. [15] among the few studies employing external validation with CBCT data, enhancing the reliability of its AI tool for lesion detection, although its real-time clinical performance remains undocumented. These examples emphasize the gap between in-lab model performance and actual clinical implementation, underscoring the need for future research to prioritize external validation, workflow integration, and long-term outcome assessment. Real-world clinical scenarios vary in complexity due to diverse patient populations, differing healthcare infrastructures, and unpredictable case presentations. Therefore, real-world usability studies are crucial to determine how AI tools integrate with existing clinical workflows, influence decision making, and affect patient outcomes. These studies also reveal adoption barriers such as clinician resistance, interoperability issues, and cost-related challenges.

Furthermore, for AI systems to be safely and legally deployed in dental practice, they must comply with stringent regulatory standards. In the United States, the Food and Drug Administration (FDA) mandates rigorous validation through clinical trials for approval of AI-based medical tools [36]. Similarly, in the European Union, AI technologies must conform to the Medical Device Regulation (MDR) and obtain CE marking [37]. To date, only a limited number of dental AI tools have received such regulatory clearance, underscoring the need for stronger governance and transparency in AI development. Long-term, multicenter trials are necessary to assess the sustained performance, safety, and ethical implications of AI systems in diverse dental care settings.

Although initial implementation costs—including software acquisition, hardware infrastructure, staff training, and data security compliance—pose challenges, AI can still be a cost-effective solution in the long term. Scalable and subscription-based AI models, particularly those requiring minimal hardware, are becoming increasingly available. These innovations may make AI adoption feasible even for small dental practices, provided that barriers such as clinician training and workflow compatibility are adequately addressed.

## 5. Limitations

Despite the promising outcomes identified in this systematic review, several limitations must be acknowledged. First, many of the included studies relied on datasets from limited or homogenous populations, potentially affecting the generalizability of their findings. For example, studies such as those by Patcas et al. [12,18] and Yamaguchi et al. [11] focused on specific geographic regions or institutional datasets without external validation, limiting their broader applicability.

Second, while the diagnostic accuracy of AI models like CNNs and hybrid neural networks (e.g., LB-ABC BPNNs) was consistently high in controlled settings [4,14,16], few studies validated their performance in real-world clinical environments. The absence of external validation in several studies, such as Arisu et al. [6] and Kuwada et al. [10], raises concerns about the robustness and reproducibility of the models when applied across diverse clinical contexts.

Third, reporting inconsistencies were noted across studies, particularly regarding key methodological elements like data preprocessing, sample size estimation, and validation strategies. For instance, validation methods were either not clearly stated or limited to internal testing in studies by Li et al. [8], Lee et al. [20], and others, potentially impacting the reliability of the reported outcomes.

Finally, most reviewed studies did not assess long-term performance or model adaptability over time. Real-world deployment demands continuous learning and validation of AI systems across varying datasets, clinical workflows, and patient demographics. This calls for future longitudinal, multicenter investigations to evaluate the sustained efficacy, ethical implications, and integration feasibility of AI tools in diverse dental practice settings.

## 6. Conclusions

AI represents a potent and efficacious tool in the field of dentistry, characterized by high diagnostic specificity, sensitivity, and accuracy. These attributes render AI valuable in augmenting diagnostic processes and, more broadly, enhancing patient care. Nevertheless, to substantiate these findings, it is imperative to incorporate large sample sizes. Future research should focus on evaluating the effectiveness and therapeutic interventions facilitated by AI models in dental care.

In conclusion, the prospective integration of artificial intelligence into clinical practice necessitates the implementation of long-term, large-scale multicenter randomized controlled trials to comprehensively evaluate its efficacy, particularly across diverse patient demographics. Establishing minimum benchmarks for comparing AI performance is a crucial step towards building a robust foundation for AI applications in dentistry.

Artificial intelligence and machine learning hold the potential to become significant assets in clinical dentistry, enhancing the efficacy of diagnostic processes and potentially increasing the accuracy of primary diagnoses. However, their widespread applicability and optimal utilization can only be realized through substantial investment in further research and technological advancements. As these technologies evolve, they may facilitate the prompt diagnosis and treatment of young patients within new dental practices, thereby improving clinical outcomes.

## Figures and Tables

**Table 2:** Evaluations of methodological quality included studies.

Study (Author, Year)	Randomization	Blinding	Withdrawal/Dropout Mentioned	Multiple Variables Measurement	Estimation of Sample Size	Clear Exclusion/Inclusion Criteria	Reliability of Examiner	Prespecified Outcomes	Study Quality/Bias Risk
Bouchahma et al., [4] (2019)	Not conducted	Implemented	Mentioned	Measured Repeatedly	Estimated	Clearly Defined	Tested	Predetermined	Low
Arisu et al., [6] 2018	Not Conducted	Not Applied	Mentioned	Measured Repeatedly	Estimated	Clearly Defined	Tested	Not Specified	Moderate
Takahashi et al., [7] 2020	Not Conducted	Not Applied	Mentioned	Measured Repeatedly	Estimated	Clearly Defined	Tested	Predetermined	Low
Li et al., [8] 2020	Not Conducted	Not Applied	Mentioned	Measured Repeatedly	Estimated	Clearly Defined	Tested	Predetermined	Low
Kuwada et al., [10] 2020	Conducted	Not Applied	Mentioned	Measured Repeatedly	Estimated	Clearly Defined	Tested	Predetermined	Low
Yamaguchi et al., [11] 2019	Not conducted	Not Applied	Unclear	Measured repeatedly	Estimated	Clearly Defined	Tested	Not Specified	Moderate
Patcas et al., [12] 2019	Not Conducted	Not Applied	Mentioned	Measured Repeatedly	Estimated	Clearly Defined	Tested	Predetermined	Low
Li et al., [13] (2015)	Unclear	Unclear	Mentioned	Measured Repeatedly	Estimated	Clearly Defined	Not Tested	Predetermined	Moderate
Kositbowornchai et al., [17] (2016)	Not Conducted	Not Applied	Mentioned	Measured Repeatedly	Estimated	Clearly Defined	Tested	Predetermined	Low
Patcas et al., [18] (2019)	Not Conducted	Not Applied	Mentioned	Measured Repeatedly	Estimated	Clearly Defined	Tested	Predetermined	Low
Vranckx et al., [19] (2020)	Conducted	Not Applied	Mentioned	Measured Repeatedly	Estimated	Clearly Defined	Tested	Predetermined	Moderate
Lee et al., [20] (2020)	Not Conducted	Not Applied	Mentioned	Not Measured Repeatedly	Estimated	Not clearly Defined	Tested	Not specified	Moderate
Cui et al., [21] (2020)	Not Conducted	Not Applied	Mentioned	Measured Repeatedly	Estimated	Not Clearly Defined	Tested	Not Specified	Low
Sornam and Prabhakaran [16] (2019)	Not Conducted	Not Applied	Mentioned	Measured Repeatedly	Estimated	Clearly Defined	Tested	Predetermined	Low
Setzer et al., [15] (2020)	Not Conducted	Not Applied	Mentioned	Measured Repeatedly	Estimated	Clearly Defined	Tested	Predetermined	Low
Cantu et al., [14] (2020)	Not Conducted	Not Applied	Mentioned	Measured Repeatedly	Estimated	Clearly Defined	Tested	Predetermined	Low
Aliaga et al., [22] (2020)	Not Conducted	Not Applied	Mentioned	Measured Repeatedly	Estimated	Clearly Defined	Tested	Predetermined	Low
Kim et al., [23] (2018)	Not Conducted	Not Applied	Mentioned	Measured Repeatedly	Estimated	Clearly Defined	Tested	Predetermined	Low
Dumast et al., [24] (2018)	Not Conducted	Not Applied	Mentioned	Measured Repeatedly	Estimated	Clearly Defined	Tested	Predetermined	Low
Sorkhabi and Khajeh [25] (2019)	Not Conducted	Not Applied	Mentioned	Measured Repeatedly	Estimated	Clearly Defined	Tested	Predetermined	Low

**Table 3:** Quality assessment results.

Design of Study	No. of Studies	Percentage	Bias Risks
Randomized	2	76	Moderate
Cross-sectional	9	80	Low
Diagnostic test accuracy	9	88	Low

Low = low risk/concern; Moderate = some concerns.

**Table 4:** Comparative overview of AI models used in dental diagnostics.

AI Model	Diagnostic Accuracy	Interpretability	Deployment Feasibility	Suitable Applications
CNN	High (85–93%)	Low	Requires GPU resources	Image-based diagnostics (caries, lesions)
Hybrid BPNN (LB-ABC)	Very High (~99%)	Moderate	Moderate complexity	Oral cancer, caries classification
DNN	High to Very High	Low	Moderate to High	Treatment planning, structured data tasks

## Data Availability

The dataset used in this paper will be made available upon request to the corresponding author.
